# A Case Report of a Mature Omental Teratoma: Insights Into Extragonadal Teratoma Formation and Management

**DOI:** 10.1155/cris/9926848

**Published:** 2026-06-17

**Authors:** Mohamad Behzadi, Nazanin Anaraki, Javad Salimi

**Affiliations:** ^1^ General Surgery Department, Sina Hospital, Tehran, Iran; ^2^ Surgery Department, Tehran University of Medical Sciences, Tehran, Iran, tums.ac.ir

**Keywords:** case report, mature teratoma, omental teratoma, surgery, teratoma

## Abstract

Teratomas are germ cell tumors, with ovarian teratomas being the most common type. However, extragonadal teratomas are extremely rare. Omental teratomas are the most common type of extragonadal teratomas. Most patients with omental teratomas are asymptomatic, making preoperative diagnosis challenging. Imaging studies, such as CT scans, can provide valuable information, but a definitive diagnosis requires histopathological examination. In this case, a 65‐year‐old Iranian woman presented with mild abdominal pain and a normal physical examination. A preoperative abdominopelvic CT scan revealed a calcified pelvic mass with fat components, suggesting a connection to the right fallopian tube. However, during surgery, the mass was found to be in the greater omentum, with no adnexal involvement. Histopathological examination confirmed a mature teratoma. Treatment for mature teratomas typically involves surgical excision, which was successfully performed in this case, resulting in a smooth postoperative recovery.

## 1. Introduction

Teratomas are germ cell tumors that typically arise in the gonads and contain elements from all three germinal layers [1]. Teratomas are classified into mature and immature teratomas. Mature teratomas are usually benign and are mostly located in the ovaries [2–4]. However, extragonadal teratomas, such as those in the omentum, are extremely rare. The incidence of teratomas is significantly lower in men compared to women [5, 6]. Preoperative diagnosis of teratomas is not easy, and the diagnosis is confirmed after excision and biopsy results. The proper treatment for mature teratomas is surgical excision. This case report discusses a mature omental teratoma in a middle‐aged Iranian woman that mimicked an ovarian teratoma before surgery. This teratoma was successfully treated via surgical excision.

## 2. Case Presentation

A 65‐year‐old Iranian woman with a 1‐year history of mild, dull abdominal pain was referred to the general surgery department. She was postmenopausal with a history of six normal vaginal deliveries. The pain was nonradiating, did not follow any specific pattern, and she did not experience nausea, vomiting, or weight loss. Gas passage and defecation were normal. She had no history of surgery but reported oral opium use. General physical, abdominal, and systemic examinations were unremarkable, with no signs of hepatomegaly or ascites. Hematologic, coagulation, and biochemical parameters, including tumor marker tests (CA125, CA19‐9, carcinoembryonic antigen, and alpha‐fetoprotein), were all within normal limits. A plain abdominal X‐ray revealed a large calcified mass in the pelvis (Figure 1).

**Figure 1:**
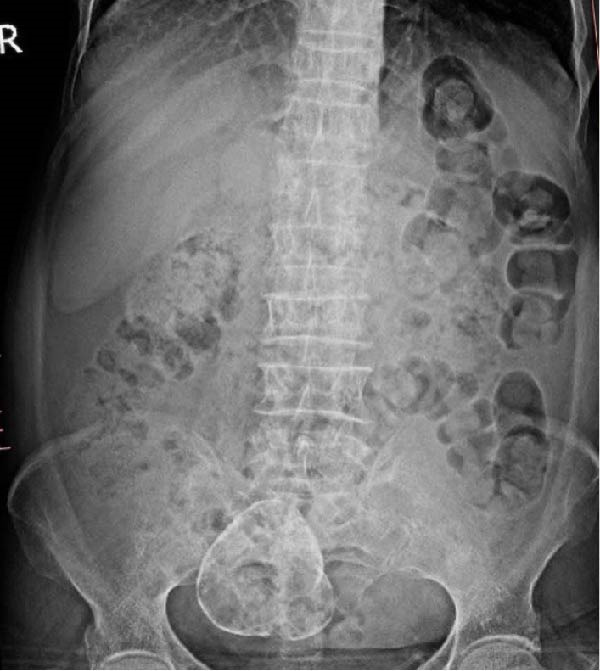
A plain abdominal radiograph demonstrating a well‐defined, rounded, heterogenous soft‐tissue density mass in the pelvic region, containing internal calcific components.

An abdominal CT scan with IV and oral contrast showed a pelvic mass with peripheral thick eggshell wall calcification and internal mixed soft tissue and fat densities, measuring 65 mm × 45 mm, appearing to be connected to the right fallopian tube. The differential diagnosis suggested a mature ovarian teratoma, peritoneal infarction, and, less likely, a gastrointestinal stromal tumor (GIST; Figure 2). A mature ovarian teratoma was initially suspected due to the presence of fat and calcification within the pelvic mass and its apparent connection to the right fallopian tube on CT imaging. Peritoneal infarction was considered because of the mass’s location adjacent to the peritoneal folds and the presence of peripheral calcification, which can occur in chronic infarction. GIST was deemed less likely, as these tumors usually present with soft tissue density without fat components; however, it was included in the differential due to the mass’s size and mixed density pattern.

**Figure 2:**
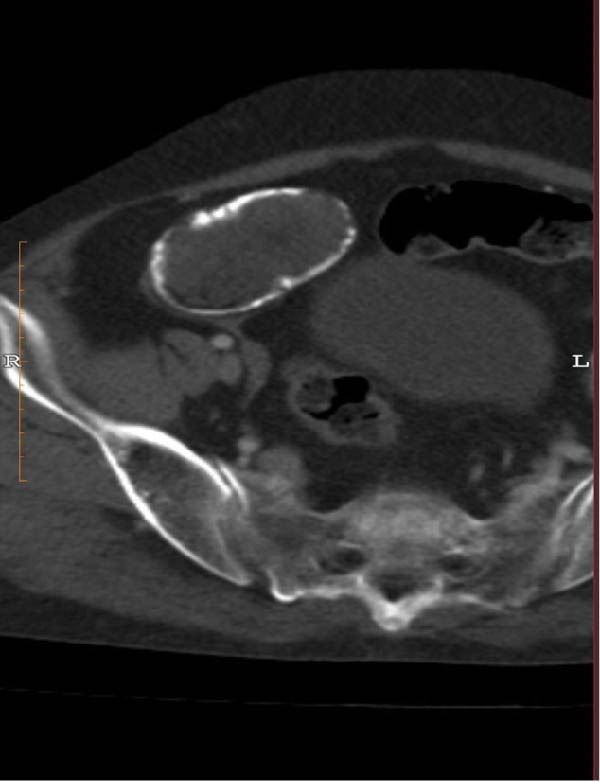
Contrast‐enhanced abdominal CT scan showing a pelvic mass (65 mm × 45 mm) with peripheral thick eggshell wall calcification and internal mixed soft tissue and fat densities, appearing to be connected to the right fallopian tube. The differential diagnosis included mature teratoma (dermoid cyst), peritoneal infarction, and, less likely, gastrointestinal stromal tumor (GIST).

After discussing the situation with the patient, a midline laparotomy was planned. Intraoperative findings revealed an atrophic uterus and ovaries. Contrary to the CT report, there was no connection between the teratoma and the fallopian tube; instead, the teratoma was located in the greater omentum (Figure 3). The consistency of the teratoma’s exterior was firm but brittle, and after opening it, abundant hair was found inside (Figure 4). The surgeon found the small and large intestines to be normal, with no palpable pelvic or paraaortic lymph nodes. The mass was excised through total omentectomy, and a peritoneal wash for cytology was collected. The pathology report confirmed the presence of a mature teratoma, with histopathological examination revealing well‐differentiated tissues derived from the ectodermal and mesodermal germ layers, including hair follicles, muscle tissue, and connective tissue (Figure 5). The patient experienced a smooth postoperative recovery.

**Figure 3:**
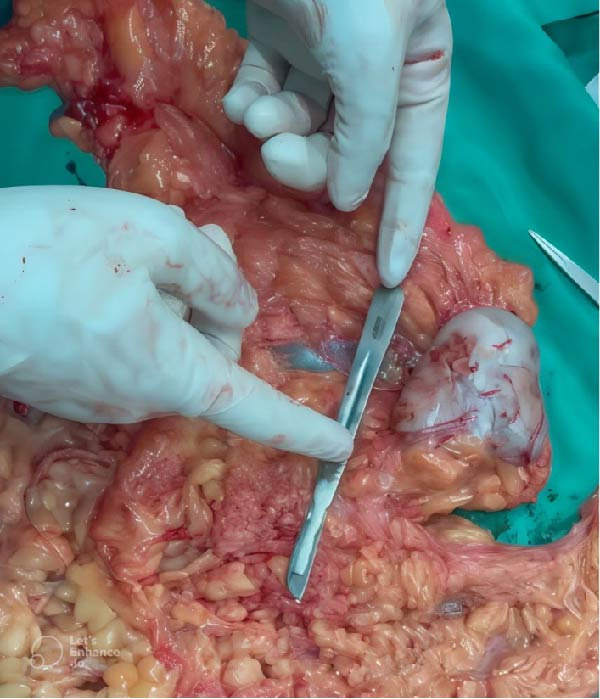
Intraoperative image showing the mature teratoma located within the greater omentum, with no connection to the adnexa, highlighting the discrepancy with preoperative CT findings.

**Figure 4:**
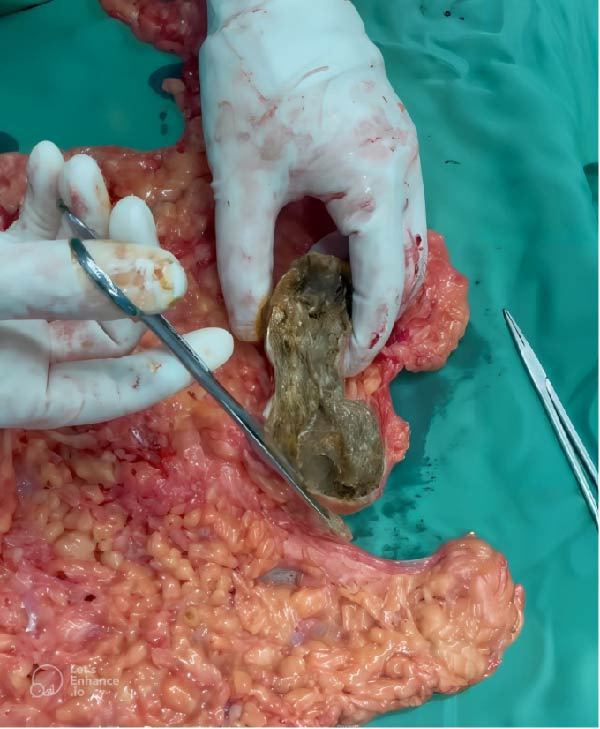
Intraoperative view of the excised mature omental teratoma after sectioning, revealing abundant hair within the cystic cavity.

**Figure 5:**
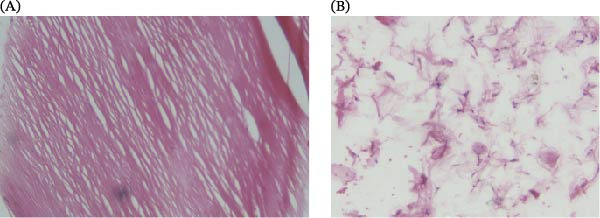
H&E‐stained sections demonstrating (A) dense fibrous connective tissue composed of parallel collagen bundles and (B) mature adipose tissue separated by delicate fibrous septa. No cytologic atypia or immature elements are identified. These images illustrate selected tissue components identified in the pathology report and are not intended to represent the full spectrum of differentiated elements of the lesion.

## 3. Discussion

Teratomas are tumors originating from germ cells. Based on the level of differentiation of the tissue components, teratomas are classified into mature and immature teratomas. Immature teratomas are malignant and can replicate the differentiation traits of fetal tissue. They contain varying amounts of immature tissues, primarily composed of primitive tissues [7].

Mature teratomas are benign and are predominantly located in the ovaries. Over 95% of teratomas are represented by mature cystic ovarian teratomas, commonly referred to as “dermoid cysts” [1]. However, extragonadal teratomas are extremely rare [2]. Omental teratomas are the most frequently reported extragonadal teratomas and typically occur in women. While they are usually found in women of reproductive age, they can also appear in young girls and older women [8]. Extragonadal teratomas can also occur in other locations, such as the pouch of Douglas, liver, and inguinal canal [8].

Extragonadal teratomas located within the peritoneum are sometimes referred to as “wandering teratomas” due to their ability to change position and move around within the pelvic region [9]. This tumor has also been described using other terms, such as “parasitic teratoma,” due to its tendency to draw blood from native tissues at its ectopic implantation sites [10, 11]. As the tumor gradually loses its own independent blood supply, it starts to attach to adjacent tissues and establishes collateral connections with the existing blood vessels, effectively leeching blood from the surrounding structures [10].

The exact causes of extragonadal teratomas remain unclear, but three primary theories have been suggested:

1. Omental teratomas may develop from germ cells that are displaced during early fetal development. Normally, germ cells migrate from the yolk sac to the reproductive crest, but in this case, an error occurs, causing them to become trapped between the yolk sac and dorsal mesentery [11–13].
2. Teratomas may arise from an extra (supernumerary or accessory) ovary located in the omentum. Supernumerary ovaries are entirely separate from the normal ovaries and originate from a single primordial structure. Accessory ovaries, on the other hand, consist of multiple ovarian tissues found near the normal ovaries and may be connected to or derived from them. In embryology, this is linked to the migration of primitive cells from the yolk sac to the gonadal ridge, potentially due to delayed germ cell migration [12, 14, 15].
3. Teratomas may also develop from an auto‐amputated ovarian dermoid cyst that subsequently implants into the greater omentum, often as a result of chronic or acute torsion. This mechanism is the most frequently reported in various studies and case reports [12, 15, 16].

The symptoms of extragonadal teratomas vary depending on their location. In most cases, patients with omental teratomas are asymptomatic, and these teratomas are often discovered incidentally [3, 17]. However, abnormal uterine bleeding or a vaginal mass may occur in postmenopausal women with teratomas located in the uterus, cervix, or ovarian tube. Those with teratomas in the pouch of Douglas may experience hypogastric pain, though some patients may not have any pain or noticeable symptoms at all [7, 18].

The presence of fat and calcification is a key finding in preoperative diagnosis, making CT scans the preferred diagnostic tool. In addition, imaging plays a crucial role in differentiating teratomas from malignant gynecologic conditions, particularly uterine sarcomas, which may present with similar clinical manifestations in postmenopausal women. Ultrasonography with Doppler assessment and magnetic resonance imaging can provide important complementary information for this differential diagnosis, as imaging characteristics such as vascular patterns, tissue heterogeneity, and lesion borders may raise suspicion for malignancy [19, 20]. A definitive diagnosis, however, is only established through histopathological evaluation [21]. In the present case, preoperative imaging suggested a possible connection between the mass and the fallopian tube, whereas intraoperative findings revealed that the tumor was located in the greater omentum with no adnexal attachment. This discrepancy highlights the limitations of imaging and underscores the critical role of intraoperative assessment in accurately identifying tumor location and guiding surgical management. The presence of hair and calcification within the excised mass strongly suggested a teratoma, which was subsequently confirmed histopathologically.

The treatment of teratomas is determined by their degree of maturity. For mature teratomas, surgical removal is usually sufficient. However, immature teratomas are considered potentially malignant and may require postoperative chemotherapy and radiotherapy [21] (Table 1).

**Table 1:** Summary of learning points.

Learning points	Description
Rarity of extragonadal teratomas	Extragonadal teratomas, particularly those located in the omentum, are extremely rare compared to ovarian teratomas, which are more common.
Diagnostic challenges	Preoperative diagnosis of extragonadal teratomas is challenging due to the limitations of imaging studies in accurately identifying them.
Histopathological confirmation	Definitive diagnosis of teratomas requires histopathological examination following surgical excision.
Management of mature teratomas	The primary treatment for mature teratomas is surgical excision.
Potential mechanisms of extragonadal teratomas formation	Three theories explain the development of extragonadal teratomas: germ cell displacement, the presence of accessory ovaries, and the auto‐amputation of ovarian teratomas
Role of intraoperative findings	Intraoperative assessment provides definitive information about the tumor’s location, extent, and relationship to adjacent structures, and can clarify or complement preoperative imaging findings, thereby guiding appropriate surgical management.

This case emphasizes the rarity and diagnostic complexity of extragonadal teratomas and highlights the importance of considering them in the differential diagnosis of pelvic and abdominal masses. Careful intraoperative evaluation is essential to confirm tumor origin and characteristics, particularly when preoperative imaging is inconclusive. Reporting such rare cases contributes to the limited existing literature and may improve awareness, diagnostic accuracy, and surgical planning for similar cases in the future.

## Funding

No funding was received for this study.

## Consent

The patient provided written informed consent for publication of this case report and any accompanying images.

## Conflicts of Interest

The authors declare no conflicts of interest.

## Data Availability

The data that support the findings of this study are available from the corresponding author upon reasonable request.
